# Enhanced performance of direct-current triboelectric nanogenerators based on SiO_2_-PVDF composite fibers coated with TiO_2_*via* spatial atomic layer deposition

**DOI:** 10.1039/d6ra03149h

**Published:** 2026-07-03

**Authors:** Duy Linh Vu, Thi Thuong Nguyen, Dinh Nam Nguyen, Hung-Anh Tran Vu, Ha Thi Vu Nguyen, Quang Tan Nguyen, Nguyen Xuan Duong, Ngoc Thanh Duong, Hieu Minh Nguyen, Viet Huong Nguyen

**Affiliations:** a Faculty of Materials Science and Engineering, Phenikaa School of Engineering, Phenikaa University Hanoi 12116 Vietnam linh.vuduy@phenikaa-uni.edu.vn huong.nguyenviet@phenikaa-uni.edu.vn; b Center for Environmental Intelligence, VinUniversity Hanoi 100000 Vietnam; c Faculty of Electronics and Telecommunications, VNU University of Engineering and Technology, Vietnam National University Hanoi 12116 Vietnam

## Abstract

Direct-current triboelectric nanogenerators (DC-TENGs) based on metal–semiconductor interfaces are attractive for self-powered electronics because they can generate usable direct current without external rectification. However, their performance is often limited by insufficient charge transport in porous polymer membranes, especially under humid conditions. Herein, we report a DC-TENG based on SiO_2_-PVDF composite fibers fabricated *via* vapor phase infiltration (SiO_2_-VPI) and subsequently coated with TiO_2_ by spatial atomic layer deposition (TiO_2_-SALD). Porous PVDF fibers membranes were first prepared by electrospinning a PVDF/PVP precursor, followed by PVP removal. SiO_2_-VPI was then employed to incorporate SiO_2_ into the PVDF fiber network, forming SiO_2_-PVDF composite membrane while preserving the porous morphology. This treatment improved the membrane hydrophilicity and promoted more uniform TiO_2_ deposition during the subsequent SALD process. As a result, the optimized membrane exhibited significantly enhanced DC-TENG performance under humid conditions, delivering a current density of 341 µA cm^−2^ and an output voltage of 0.72 V. A maximum power density of 82.8 µW cm^−2^ was obtained at 3000 Ω, which is approximately twice that of the direct-TiO_2_-SALD control. The device also showed stable operation over 4000 cycles and was capable of directly charging capacitors and powering a digital thermo-hygrometer. These results demonstrate that the integration of SiO_2_-PVDF composite fibers with TiO_2_ coating by SALD provides an effective route to high-performance DC-TENGs for energy harvesting.

## Abstract 2

SiO_2_ vapor-phase infiltration (VPI) activates the interior of porous PVDF fiber membranes, while spatial atomic layer deposition (ALD) forms a conformal TiO_2_ shell to create a humidity-responsive, Schottky-regulated pathway for DC-TENGs.

## Graphical abstract

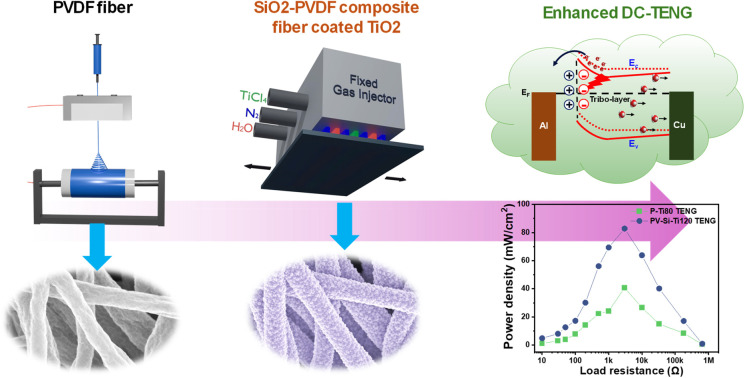

## 1 Introduction

The growing demand for wearable electronics, distributed sensors, and self-sustained microsystems has intensified the search for lightweight technologies capable of harvesting mechanical energy from the environment. Among the available approaches, triboelectric nanogenerators (TENGs) have emerged as a highly attractive platform because of their simple device architecture, broad materials compatibility, low cost, and excellent performance under low-frequency mechanical excitation.^1–5^ These advantages make TENGs promising for converting irregular energy sources, such as human motion, vibration, and airflow, into usable electrical output. However, most conventional TENGs inherently generate alternating current (AC), which typically requires external rectification before practical use in powering electronics or charging energy-storage units.^6–9^ This added circuitry increases system complexity, introduces conversion loss, and limits device miniaturization and integration.

To overcome these limitations, direct-current (DC) mechanical energy harvesters based on asymmetric interfacial charge transport have attracted increasing attention.^10,11^ In particular, metal–semiconductor contacts can promote directional carrier extraction during contact electrification or interfacial friction, enabling native DC output without external rectifiers. Compared with AC-type TENGs, these DC nanogenerators are intrinsically advantageous for compact power systems because they simplify circuit design and improve output usability.^12–14^ Nevertheless, the realization of high-performance DC nanogenerators remains challenging, especially under humid conditions, where environmental water strongly influences charge generation, transport, and retention.^8,15,16^ Humidity is generally considered detrimental to triboelectric output because adsorbed water can screen surface charges, accelerate recombination, and increase leakage pathways. As a consequence, most triboelectric devices suffer pronounced signal decay and poor operational stability at high relative humidity. Yet the role of water in interfacial energy conversion is more complex than a simple loss mechanism. When materials and interfaces are rationally designed, absorbed moisture can facilitate ionic or proton-assisted transport, enhance local polarization, and even modulate charge extraction across heterogeneous junctions.^17–19^ This creates an important opportunity: humidity can potentially be transformed from a parasitic factor into a functional stimulus for DC generation and self-powered sensing. Achieving this transition, however, requires a material system in which both the semiconductor interface and the membrane interior are deliberately engineered.

Poly(vinylidene fluoride) (PVDF) is an attractive material for such a design because of its strong electronegativity, chemical stability, flexibility. In addition, the polar nature of PVDF has made it one of the most widely investigated polymers in triboelectric, piezoelectric, and hybrid nanogenerators.^20–22^ However, pristine PVDF membranes are still limited by their hydrophobic nature and chemically passive interior, both of which restrict effective interaction with environmental moisture and limit controlled transport under humid conditions. A practical route to impart DC-generating capability to PVDF is to introduce a semiconductor oxide layer that forms a rectifying contact with a metal electrode.^23–26^ Among the available oxides, TiO_2_ is particularly attractive because of its semiconducting nature, chemical stability, environmental benignity, and compatibility with vapor-phase processing.^27,28^ Spatial atomic layer deposition (SALD) is especially suited for coating electrospun fiber membranes, as it offers excellent conformality, precise thickness control, and uniform coverage on high-aspect-ratio porous substrates. When deposited onto PVDF fibers, TiO_2_ can act as a conformal semiconductor shell that defines the metal–semiconductor interface and promotes Schottky-regulated directional charge transport, thereby supporting tribovoltaic DC generation.^29–32^ However, like conventional ALD, SALD remains primarily a surface-directed technique. Although it precisely engineers the fiber exterior, the internal polymer matrix remains largely unmodified. As a result, SALD-TiO_2_/PVDF membranes may provide a rectifying interface, but they still lack a membrane interior specifically designed to utilize humidity in a constructive manner. This limitation suggests that surface semiconductor engineering alone is insufficient for maximizing DC output in humid environments.

Vapor phase infiltration (VPI) provides an effective means to overcome this limitation by enabling vapor precursors to diffuse into the polymer matrix and react throughout subsurface and internal regions, rather than only modifying the outer surface. As a result, the fibrous membrane interior is converted from an electrically inert support into a humidity-responsive transport medium.^33,34^ When combined SALD, this creates a dual-scale design strategy for humidity-enhanced tribovoltaic DC nanogenerators: VPI activates the membrane interior for water-assisted charge transport, while SALD forms a conformal TiO_2_ shell that establishes the Schottky-regulated interface required for directional DC extraction. This internal–external coupling is fundamentally different from conventional oxide-coated polymer membranes, in which only the surface is functionalized and the bulk remains passive.^35,36^ By assigning complementary roles to the infiltrated interior and the TiO_2_ shell, this strategy enables environmental moisture to be utilized not as a source of degradation, but as a beneficial factor for amplified DC energy conversion.

Herein, we present a dual-scale vapor-phase engineering strategy for fabricating high-performance DC-TENGs based on SiO_2_-PVDF composite fiber membranes subsequently coated with TiO_2_*via* spatial atomic layer deposition. The novelty of this work lies in the combination of SiO_2_-VPI and TiO_2_-SALD to simultaneously regulate the internal charge-transport environment and the external semiconductor interface of porous PVDF fibers. In this architecture, SiO_2_-VPI introduces inorganic SiO_2_ into the interior of the porous PVDF fibers network, improving membrane hydrophilicity and promoting humidity-assisted charge transport, while the TiO_2_ layer deposited by SALD provides the semiconductor interface required for directional DC output. Owing to this synergistic internal and interfacial engineering, the optimized membrane exhibits enhanced electrical output and a more pronounced humidity response than the non-infiltrated control. In addition, the device shows stable operation and practical capability for capacitor charging and powering small electronics.

## 2 Material and methods

### 2.1. Chemicals and materials

Poly(vinylidene fluoride) powder (PVDF, *M*_w_ ∼534 000) and polyvinylpyrrolidone (PVP, *M*_w_ ∼350 000) were used as the polymer components. Silicon tetrachloride (SiCl_4_, 99.9%) and titanium tetrachloride (TiCl_4_, 99.9%) served as the precursors for SiO_2_ vapor phase infiltration and TiO_2_ spatial atomic layer deposition, respectively. These chemicals were obtained from Sigma-Aldrich, USA. *N*,*N*-dimethylformamide (DMF, 99%) and acetone (97%), supplied by Samchun Chemical, Korea, were used as solvents for preparing the electrospinning solution.

### 2.2. Preparation of porous PVDF fiber membranes

**Fig. 1:**
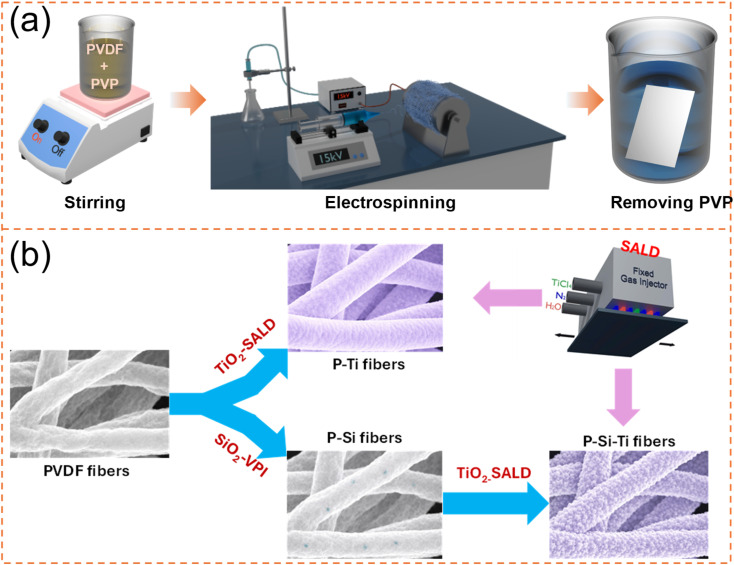
Schematic illustration of the fabrication strategy: (a) preparation of the porous PVDF membrane; (b) schematic SiO_2_-VPI treatment and TiO_2_-SALD coating.

PVDF and PVP were used to prepare porous PVDF fibers membrane by electrospinning (Fig. 1a). A PVDF/PVP precursor solution was prepared in a mixed DMF/acetone solvent, with PVP incorporated at a PVDF : PVP mass ratio of 2 : 1 to act as a removable second phase. After electrospinning, the as-spun PVDF/PVP membrane was immersed in ethanol to selectively remove PVP, yielding a wrinkled and porous PVDF membrane with enlarged surface area and improved moisture accommodation.

### 2.3. SiO_2_-VPI treatment and TiO_2_-SALD coating

Two membrane series were prepared for comparative study (Fig. 1b). In the first series, the porous PVDF fibers membrane was directly coated with TiO_2_ by SALD using 40, 80, 120, and 160 cycles. In the second series, the porous PVDF fibers membrane was first subjected to a VPI treatment using Si-containing precursors to introduce an inorganic SiO_2_-like phase into the fiber interior and junction regions, and was then coated with TiO_2_ by SALD using the same cycle numbers. The VPI process was employed to modify the membrane interior rather than only the outer surface, while the subsequent SALD process formed the conformal semiconductor shell necessary for Schottky-type transport. The nomenclature of all samples is summarized in Table 1.

**Table 1:** Nomenclature and processing conditions of porous PVDF, SiO_2_-PVDF composite fibers, and TiO_2_-coated membrane series

Sample ID	Membrane type	SiO_2_-VPI	TiO_2_-SALD (cycles)
PVDF	Porous PVDF fibers membrane	No	0
P–Si	SiO_2_-PVDF composite fibers	Yes	0
P–Ti40	PVDF fibers directly coated by TiO_2_-SALD	No	40
P–Ti80	PVDF fibers directly coated by TiO_2_-ALD	No	80
P–Ti120	PVDF fibers directly coated by TiO_2_-ALD	No	120
P–Ti160	PVDF fibers directly coated by TiO_2_-ALD	No	160
P–Si–Ti40	SiO_2_-PVDF composite fibers coated by TiO_2_-SALD	Yes	40
P–Si–Ti80	SiO_2_-PVDF composite fibers coated by TiO_2_-SALD	Yes	80
P–Si–Ti120	SiO_2_-PVDF composite fibers coated by TiO_2_-SALD	Yes	120
P–Si–Ti160	SiO_2_-PVDF composite fibers coated by TiO_2_-SALD	Yes	160

### 2.4. Device assembly and characterization

For device fabrication, the membrane was attached to a Cu electrode, while Al served as the counter electrode. The overlap between the two electrodes defined an active contact area of 1.5 × 1.5 cm^2^. Electrical outputs were measured under periodic contact-separation using a programmable mechanical actuator and a digital electrometer (DMM7510, Keithley). For humidity-related measurements, the assembled device was placed in a 100% relative humidity environment for 5 h to allow the membrane to reach maximum moisture uptake before testing.

The morphology of the prepared fiber membranes was examined using field-emission scanning electron microscopy (FE-SEM; Apreo 2 SEM, Thermo Scientific). Elemental composition and elemental distribution were analyzed by energy-dispersive X-ray spectroscopy (EDX) attached to the SEM system. Fourier transform infrared spectroscopy (FTIR; Varian 640-IR FTIR Spectrometer) was employed to analyze the chemical structure of the membranes over a spectral range of 400–4000 cm^−1^. Membrane wettability was evaluated through water contact-angle measurements using a SmartDrop system (FemtoFAB). The I–V behavior of the membrane/electrode configuration was measured using a CS350M EIS potentiostat.

## 3 Results and discussion

### 3.1. Characteristics of porous PVDF fiber membranes

**Fig. 2:**
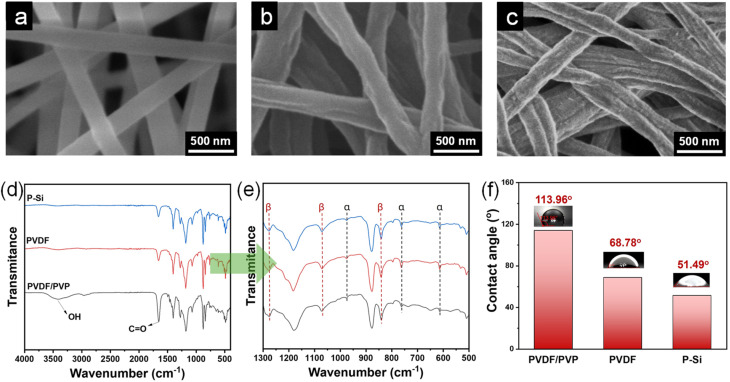
Structural and chemical evolution of the base membrane: SEM image of the (a) as-spun PVDF/PVP fibers; (b) PVDF fibers after PVP removal; (c) SiO_2_-PVDF composite fibers; (d ands e) FTIR spectra and (f) contact angle of the corresponding fibers membranes.

The morphological evolution of the base membrane is shown in Fig. 2a–c. The electrospun PVDF/PVP membrane exhibits a uniform, randomly distributed fiber network with smooth cylindrical, indicating stable jet formation and a homogeneous precursor solution. After removal of PVP, the remaining PVDF fiber becomes rougher and slightly wrinkled, and the fiber diameter decreases because extraction of the sacrificial component induces shrinkage and creates nanoscale voids. After SiO_2_-VPI treatment, no obvious morphological change is observed in the SEM images. The membrane still shows a well-preserved open fiber network with fiber shape, diameter, and surface texture largely comparable to those of the porous PVDF membrane before infiltration. This confirms that the VPI process does not disrupt the morphology of the electrospun scaffold, but instead converts the original PVDF fibers into SiO_2_-PVDF composite fibers through the incorporation of inorganic SiO_2_ within near-surface and subsurface regions.^33,37^ Importantly, this composite formation occurs without compromising the overall porous fibers architecture. Such a morphology-preserving modification is beneficial because it retains the porous framework needed for efficient interfacial contact while providing internal chemical functionality for subsequent water uptake and TiO_2_ growth.^38^

The FTIR spectra in Fig. 2d reveal clear chemical changes associated with removal of the sacrificial PVP phase. In the PVDF/PVP fibers, characteristic absorption bands of PVP are observed, including the broad O–H stretching band and the C=O stretching vibration. After PVP extraction, these bands are significantly weakened, confirming the effective removal of PVP from the fiber. In contrast, no noticeable difference is observed between the spectra of the PVDF and P–Si fibers, suggesting that the SiO_2_-VPI treatment does not introduce major changes in the main chemical features detectable in this spectral region. Fig. 2e shows that all three membranes exhibit the characteristic absorption bands of both the α- and β-phases of PVDF, including the α-phase bands at 612, 763, and 974 cm^−1^ and the β-phase bands at 840, 1070, and 1280 cm^−1^. The relative β-phase content, F(β), was estimated using the standard FTIR-based relation:^39^1
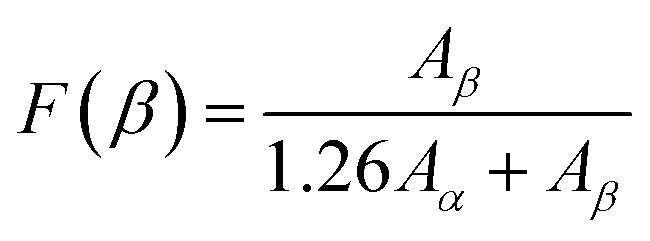
where *A*_α_ and *A*_β_ are the absorbances at 763 and 840 cm^−1^, respectively. The calculated β-phase contents are 81.0% for PVDF/PVP, 80.4% for PVDF, and 82.5% for P–Si. These values are very close to each other, indicating that neither PVP removal nor the VPI process significantly alters the crystalline phase structure of the membrane. This result confirms that pore generation and inorganic infiltration mainly modify the membrane morphology and interfacial chemistry, while preserving the crystalline of PVDF.

The wettability results in Fig. 2f are consistent with these structural and chemical changes. The PVDF/PVP membrane shows a relatively high-water contact angle of about 113.96°, indicating a nearly hydrophilic surface. After removal of PVP, the contact angle decreases sharply to 68.78°, which can be attributed to the formation of a porous structure that facilitates water penetration into the fibrous network. Following VPI-SiO_2_ treatment, the contact angle decreases further, suggesting that the infiltration process also contributes to improved surface wettability. The combined effects of porosity generation and VPI-induced chemical modification therefore enhance the hydrophilicity of the membrane, which is beneficial for subsequent humidity-assisted electrical performance.

### 3.2. Morphology of membrane after TiO_2_-based modification

**Fig. 3:**
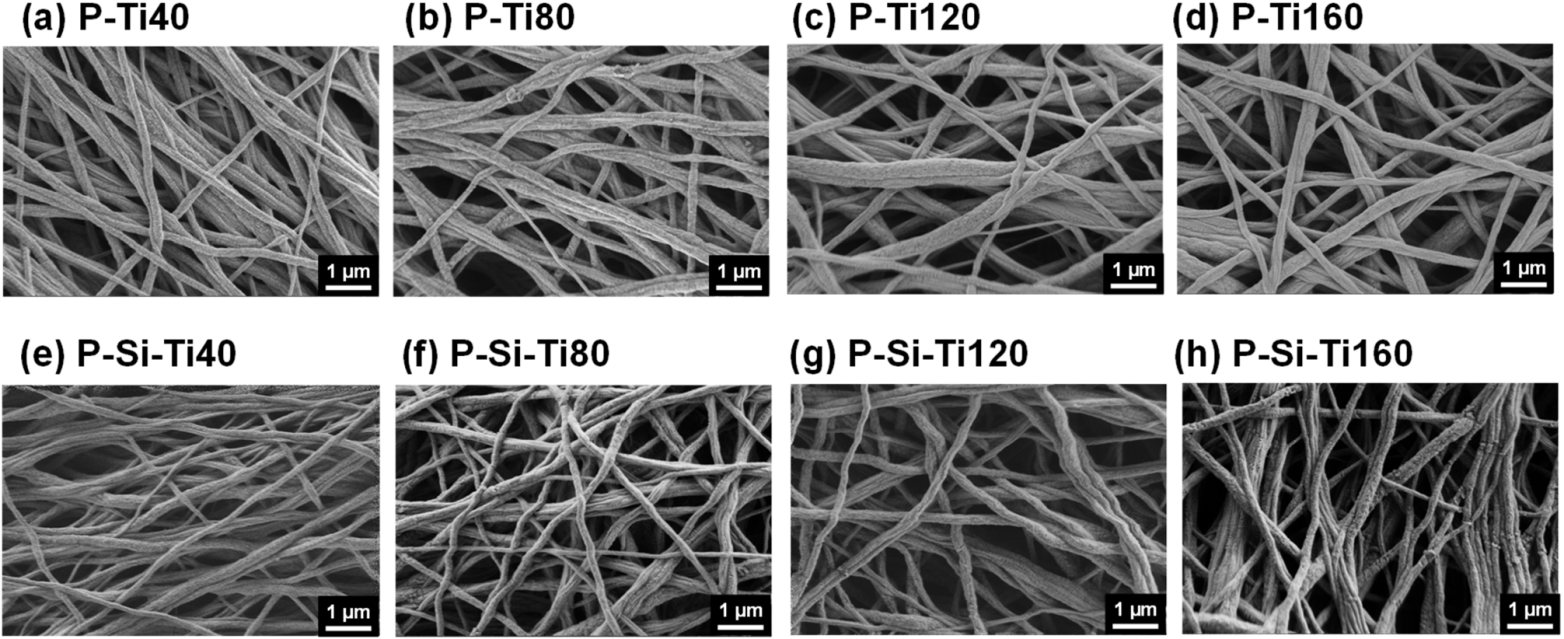
SEM images of TiO_2_-modified PVDF fibers membranes: (a–d) directly TiO_2_-SALD coated porous PVDF fibers membranes, and (e–h) SiO_2_-PVDF composite fibers membranes followed by TiO_2_-SALD.

Fig. 3 compares the SEM morphologies of the two TiO_2_-coated membrane series as a function of the number of deposition cycles. For the directly coated SALD series (Fig. 3a–d), TiO_2_ deposition at lower cycle numbers is manifested by slightly roughened fiber surfaces, while the oxide layer does not yet appear as a fully continuous shell. In this stage, the deposited TiO_2_ forms a thin conformal overlayer that follows the original fiber topography without significantly disturbing the porous membrane structure. As the number of deposition cycles increases, a more distinct and continuous TiO_2_ shell gradually develops around each fiber. The fibers exhibit a denser and more uniform outer layer, indicating the formation of a well-established conformal coating.

In contrast, the VPI treatment series (Fig. 3e–h) shows more homogeneous TiO_2_ coverage even at relatively early deposition stages. At moderate cycle numbers, the membrane already appears more uniform than its directly coated counterpart, suggesting that the prior VPI treatment improves the subsequent TiO_2_ growth behavior. This observation implies that VPI creates a more favorable chemical environment for SALD nucleation, thereby promoting earlier coalescence and more continuous oxide formation. However, at the highest cycle number, the coating becomes rougher and less uniform, indicating that excessive oxide growth may induce surface irregularities and reduce coating homogeneity. Overall, these morphological results suggest that VPI pretreatment is beneficial for achieving more uniform TiO_2_ deposition at intermediate growth stages, whereas excessive deposition cycles may compromise structural uniformity. Such a balance between coating continuity and surface roughness is likely to play an important role in determining the electrical performance of the membranes.

**Fig. 4:**
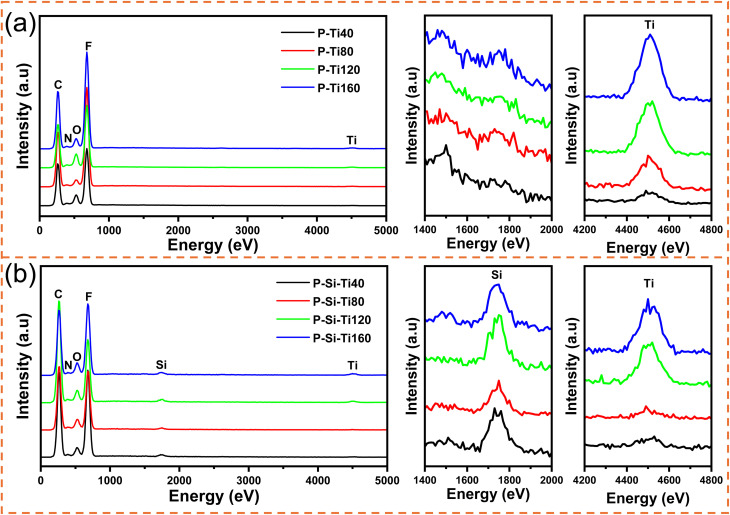
EDX spectra of TiO_2_-modified PVDF fibers membranes: (a) directly TiO_2_-SALD coated porous PVDF fibers membranes and (b) SiO_2_-PVDF composite fibers membranes followed by TiO_2_-SALD.

Elemental analysis further highlights the different roles of the two modification routes, as shown in Fig. 4 and Table 2. For the directly coated membranes (Fig. 4a), the EDX spectra reveals a gradual increase in the Ti signal with increasing numbers of TiO_2_ deposition cycles, confirming the controllable growth of TiO_2_ on the porous PVDF substrate. This trend is also reflected in the quantitative elemental composition, where the Ti content increases from 0.3 wt% for P–Ti40 to 0.5, 1.1, and 1.2 wt% for P–Ti80, P–Ti120, and P–Ti160, respectively. These results are consistent with the SEM observations, indicating that the deposited oxide progressively evolves from an ultrathin surface layer into a more continuous shell as the number of SALD cycles increases.

**Table 2:** Elemental composition of TiO_2_-SALD coated porous PVDF fibers membranes and SiO_2_-PVDF composite fibers membranes followed by TiO_2_-SALD

Sample ID	Element					
	C (wt%)	N (wt%)	O (wt%)	F (wt%)	Ti (wt%)	Si (wt%)
P–Ti40	35.6	2.2	9.8	52.1	0.3	0.0
P–Ti80	32.7	2.4	4.5	59.8	0.5	0.0
P–Ti120	32.0	2.4	6.6	57.9	1.1	0.0
P–Ti160	30.8	2.4	12.5	53.1	1.2	0.0
P–Si–Ti40	43.3	1.7	5.8	48.3	0.3	0.6
P–Si–Ti80	42.2	2.7	8.4	45.4	0.8	0.5
P–Si–Ti120	47.9	1.6	8.7	39.6	1.5	0.7
P–Si–Ti160	38.0	1.8	9.3	48.2	2.0	0.7

For the VPI treatment membranes (Fig. 4b), the EDX spectra shows the presence of both Ti and Si, confirming that the VPI process introduces a silica-containing phase prior to TiO_2_ deposition. As summarized in Table 2, the Si content remains detectable in all VPI-treated samples (0.5–0.7 wt%). To further clarify the spatial distribution of the Si-containing phase, EDS QuantMap analysis was performed for the VPI-treated membrane. As shown in Fig. S1 (SI), the Si signal is distributed along the fibrous network, supporting the successful incorporation of Si after VPI treatment. More importantly, the Ti content in the VPI and SALD membranes are consistently higher than that of the directly coated counterparts at the same thickness, increasing from 0.3 wt% in P–Si–Ti40 to 0.8, 1.5, and 2.0 wt% in P–Si–Ti80, P–Si–Ti120, and P–Si–Ti160, respectively. This result indicates that the VPI treatment facilitates subsequent TiO_2_ growth, likely by introducing chemically favorable oxygen-containing sites that promote SALD nucleation and improve oxide deposition efficiency.

### 3.3. Operating mechanism of the humidity-enhanced DC-TENG

**Fig. 5:**
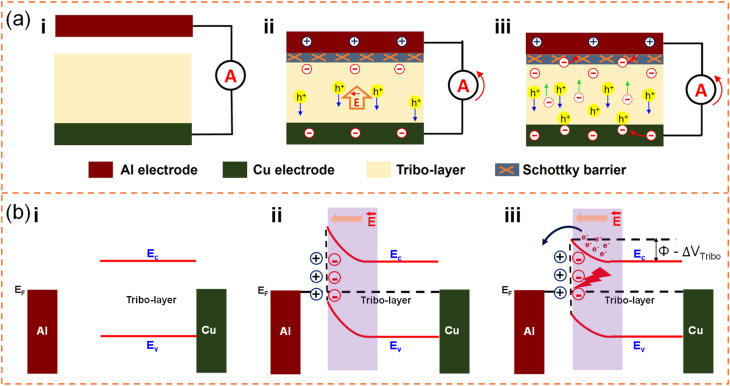
Working principle of the DC-TENG and corresponding band-diagram model showing Schottky-regulated charge extraction in the membrane/electrode system.

The operating mechanism of the TENG is illustrated in Fig. 5. The device combines a triboelectric contact process with asymmetric carrier extraction across a metal–semiconductor interface.^36^ In the initial state, before contact, the Al electrode and the tribo-layer are electrically neutral and no net current flows through the external circuit (Fig. 5a(i) and b(i)). Once mechanical contact is established, contact electrification occurs at the Al/tribo-layer interface because of the difference in their electron affinity (Fig. 5a(ii)). Meanwhile, the TiO_2_ layer forms a metal–semiconductor contact with the Al electrode, giving rise to a Schottky barrier and an internal built-in electric field (Fig. 5b(ii)).^31,40^ During compression and frictional contact (Fig. 5a(iii)), the triboelectric potential increases and perturbs the band diagram at the interface. As illustrated in the band diagram (Fig. 5b(iii)), the interfacial electric field and tribo-induced potential jointly facilitate carrier excitation and separation. Electrons are preferentially driven toward the Al side, while the opposite charges are collected through the counter electrode, generating a direct-current output. In this process, the TiO_2_ layer does not merely act as a passive coating; it functions as a semiconducting shell that regulates barrier-controlled charge extraction.

**Fig. 6:**
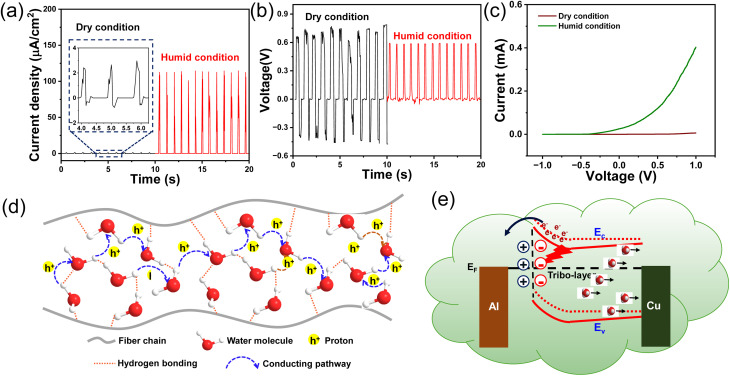
Humid-dependent electrical output of the PVDF membrane: (a) current density; (b) voltage; (c) I–V curves; schematic illustration of (d) charge-transport transport inside the PVDF membrane; and (e) proton-assisted Schottky-enhanced charge transport.

The significance of humidity is further clarified by combining the mechanism in Fig. 5 with the experimental comparison shown in Fig. 6a and b. Under dry conditions, the membrane remains resistive, and charge transport is mainly limited to the interfacial triboelectric process.^20,41^ As a result, the device exhibits only a low current density of approximately 2.4 µA cm^−2^ and a voltage of about 1.17 V in the dry state. In contrast, under humid conditions, absorbed water molecules interact with the polar sites of the membrane. This hydration process promotes the formation of hydrogen-bonded networks and facilitates proton-assisted or ion-assisted conduction throughout the membrane.^42–44^ Consequently, the membrane is transformed from a largely insulating scaffold into a humidity-activated transport medium that can support much more efficient charge migration. In these humid conditions, the current density increases dramatically to approximately 113 µA cm^−2^, which is about 47 times higher than that of the dry membrane. By comparison, the output voltage decreases to around 0.59 V. The lower output voltage under humid conditions can be attributed to the fundamentally different influence of water on charge transport and charge accumulation. The moisture screens triboelectric surface charges and accelerates charge leakage within the membrane. As a result, charges are transported and neutralized more readily rather than being retained to build up a large electrostatic potential difference. Therefore, although the wet membrane exhibits much more efficient carrier extraction and a strongly enhanced current response, the corresponding voltage decreases because the effective charge accumulation across the device is reduced under humid conditions.

The I–V characteristics in Fig. 6c further support this interpretation. Under dry conditions, the current remains close to zero throughout the applied bias range, confirming that charge injection and transport across the membrane are strongly suppressed in the absence of sufficient hydrated pathways. In contrast, the wet membrane shows a pronounced non-linear I–V response, which is characteristic of Schottky-type transport.^17^ This non-linearity indicates that, once hydrated, the membrane no longer behaves as a simple insulating dielectric layer, but instead participates in barrier-regulated charge conduction across the metal–semiconductor interface.^39,45^ To further evaluate the asymmetric charge-transport behavior, the rectification ratio was calculated using:2
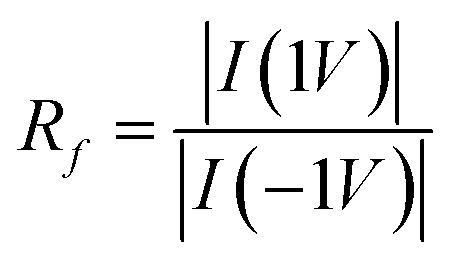


The dry membrane shows a rectification ratio of approximately 2.27, whereas the wet membrane exhibits a much higher value of about 116.72. This pronounced increase in rectification ratio indicates that humidity strongly enhances asymmetric carrier transport across the membrane/electrode interface, supporting the formation of a more effective Schottky-regulated transport pathway under humid conditions.^46^

The comparison in Fig. 6d is consistent with this transition, further demonstrating that the hydrated membrane provides a much more favorable transport environment than the dry membrane and that moisture effectively activates the internal conduction pathway required for efficient direct-current generation. Fig. 6e illustrates the mechanism of humidity-enhanced output. Under wet conditions, absorbed water molecules interact with oxygen-containing groups in the tribo-layer, promoting proton release and proton accumulation inside the membrane. This process generates an additional internal electric field, which lowers the effective Schottky barrier and facilitates carrier transport across the metal–semiconductor interface. As a result, more carriers can pass through the Schottky junction, leading to a significantly enhanced current output under humid conditions.

### 3.4. Electrical output of the TENG

**Fig. 7:**
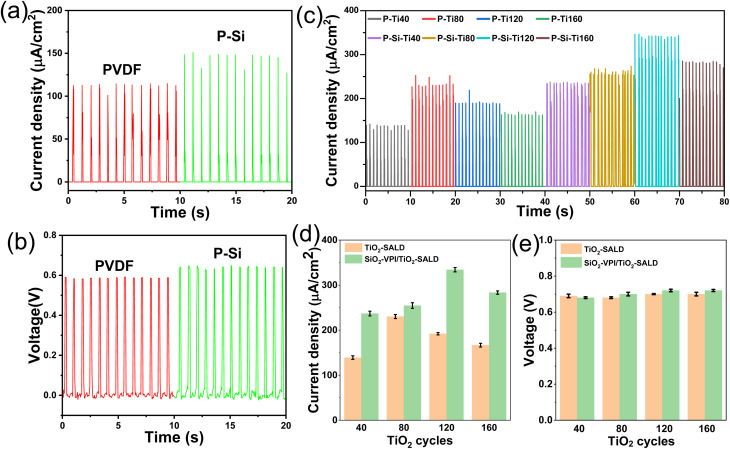
Electrical output comparison of the membrane series: (a and b) current and voltage of PVDF fibers and SiO_2_-PVDF composite fibers membranes; (c) current of P–Ti and P–Si–Ti fibers membranes with TiO_2_ cycles of 40, 80, 120, and 160; (d and e) comparison of current and voltage for representative equivalent membranes.

Fig. 7 more clearly reveals how SiO_2_-VPI treatment and subsequent TiO_2_ deposition regulate the electrical output of the device. After SiO_2_-VPI treatment, the current density increases from approximately 113 µA cm^−2^ for PVDF TENG to about 145 µA cm^−2^ for P–Si TENG, while the voltage rises from around 0.59 to 0.64 V (Fig. 7a,b). The more pronounced increase in current suggests that VPI mainly enhances charge transport through the membrane, likely by introducing a more polar and hydration-active internal environment that facilitates water-assisted carrier migration. Meanwhile, the smaller but still measurable increase in voltage indicates slightly improved charge retention. This modest voltage enhancement may be associated with the presence of the infiltrated SiO_2_ phase, which increases effective membrane resistance and suppresses rapid charge leakage.

A much greater enhancement is observed after TiO_2_ deposition, as shown in Fig. 7c. In the directly coated series, the current density first increases and then decreases with increasing numbers of TiO_2_ deposition cycles, with the P–Ti80 TENG showing the highest current density of about 230 µA cm^−2^, which is more than twice that of the pristine PVDF TENG. In the VPI treatment series, the same volcano-type trend is also observed, but with consistently higher output at matched cycle numbers. The P–Si–Ti120 TENG exhibits the highest current density among all samples, reaching approximately 341 µA cm^−2^, which is about three times higher than that of the pristine PVDF TENG. This behavior indicates that an intermediate number of TiO_2_ deposition cycles provides the best balance between forming an effective Schottky-regulated semiconducting shell and maintaining sufficiently low transport resistance through the membrane. At low cycle numbers, the TiO_2_ coating remains incomplete and the junction effect is limited; at excessively high cycle numbers, the thicker oxide shell increases the barrier width and interfacial resistance, thereby hindering carrier transfer.^36^ Moreover, after the electrodes were brought into contact without further mechanical motion, the current rapidly decreased from 341 µA cm^−2^ to approximately 25 µA cm^−2^ and then remained at this low quasi-steady level (Fig. S2, SI). This residual current, corresponding to only about 7.3% of the mechanically generated output, indicates that the galvanic-like contribution is minor. Therefore, the enhanced current in Fig. 7c is mainly attributed to mechanically activated tribovoltaic generation, Schottky-regulated charge extraction, and humidity-assisted transport.

The comparisons at matched cycle numbers in Fig. 7d and e further confirm the cooperative effect of VPI and SALD. At every deposition cycle number, the P–Si–Ti fibers membranes deliver higher current density than the corresponding P–Ti fibers membranes, clearly demonstrating that VPI makes the subsequent TiO_2_ coating more effective. The fact that the P–Si–Ti120 TENG outperforms the P–Ti80 TENG indicates that VPI treatment shifts the optimum TiO_2_ deposition condition to a higher cycle number. Without VPI, increasing the number of TiO_2_ deposition cycles 80 mainly raises the interfacial resistance and limits carrier transport. After VPI, however, the fibers membrane interior becomes more hydration-active, so the transport penalty associated with a more developed TiO_2_ shell is partially mitigated. Under this condition, a P–Si–Ti120 fibers membrane can provide a more continuous semiconducting shell and stronger Schottky-regulated charge extraction than a thinner coating. By contrast, the voltage changes only slightly across the two series, although the optimized devices can reach about 0.72 V. The limited change in output voltage suggests that VPI and SALD mainly enhance carrier transport and extraction rather than electrostatic charge accumulation. In Schottky-regulated DC-TENGs, the voltage is largely constrained by the built-in potential and interfacial barrier characteristics, whereas the current can be strongly increased through improved charge migration across the metal/semiconductor junction. Moreover, the humid environment promotes proton/ion-assisted transport but also screens surface charges, thereby suppressing high-voltage build-up.^39,47^ This result indicates that the main role of VPI is to enhance charge extraction and carrier transport rather than to dramatically increase the electrostatic potential.

**Fig. 8:**
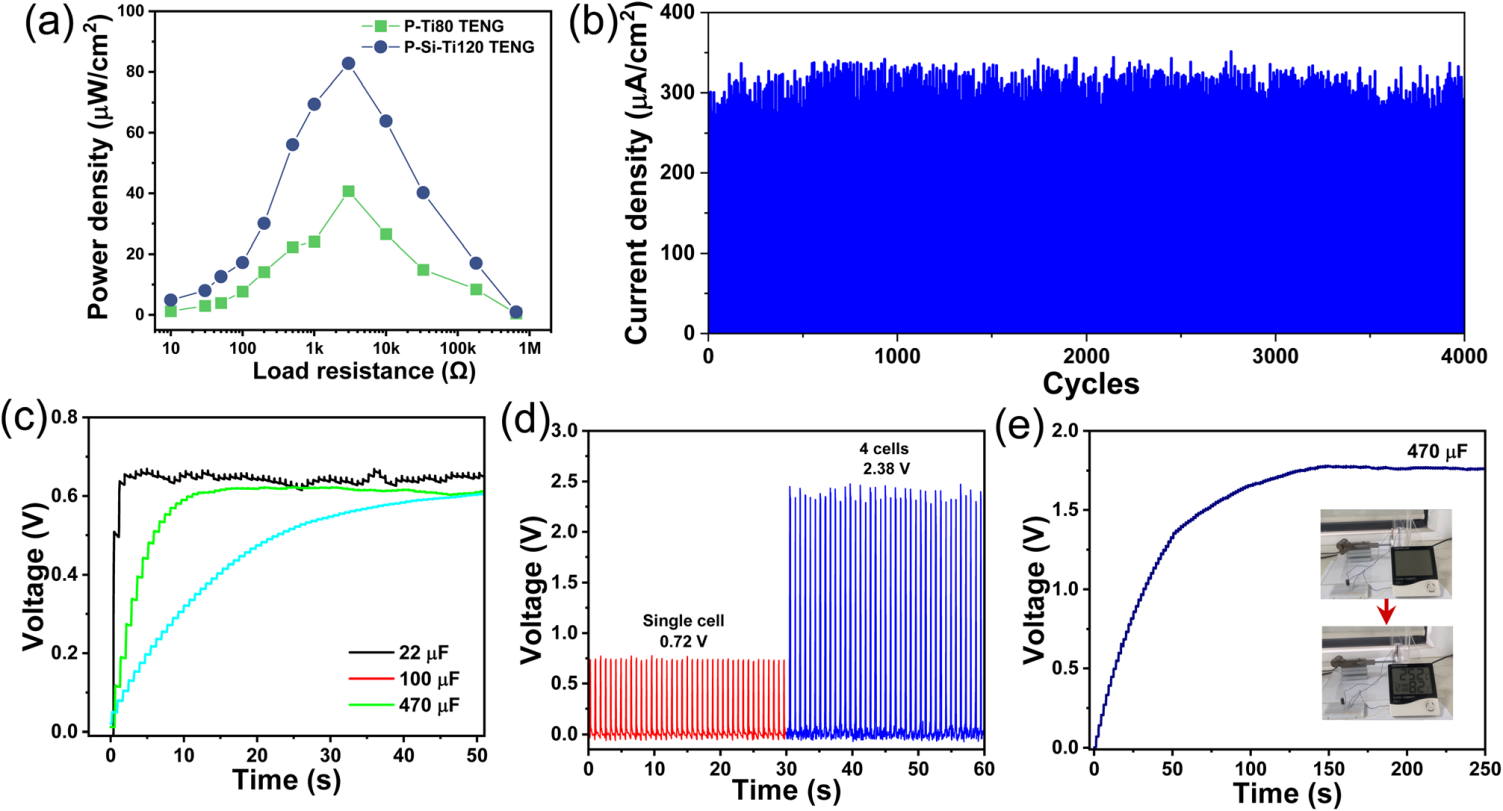
Device-level performance of the optimized membrane: (a) power density as a function of load resistance; (b) stability for 4000 cycles; (c) direct charging of a capacitor by a single TENG; (d) voltage output for a single cell and four cells connected in series; (e) charging of a 470 µF capacitor by four cells connected in series.

The optimized VPI treatment device also performs strongly at the system level, as shown in Fig. 8. In particular, the load-dependent response in Fig. 8a reveals a clear maximum in power density at an external resistance, confirming effective power delivery to a matched load rather than merely large open-circuit signals. The maximum power densities of the P–Ti80 TENG and P–Si–Ti120 TENG are 40.8 and 82.8 µW cm^−2^, respectively, and both are obtained at an external resistance of 3000 Ω. Notably, the power density of the P–Si–Ti120 device is about twice that of the directly coated P–Ti80 device, clearly demonstrating that VPI treatment substantially improves output performance beyond TiO_2_-SALD coating. This result is consistent with the earlier electrical data, where VPI enhances the internal transport environment of the membrane and enables more efficient utilization of the Schottky-regulated TiO_2_ shell. A comparison with previously reported PVDF-based TENGs further highlights the performance level of the present system. As summarized in Table 3, the P–Si–Ti120 device delivers a much higher power density than many representative PVDF-based tribo-layers. Although its power density remains lower than that of some advanced hybrid systems such as P(VDF-*co*-HFP)/MXene and PVDF/PAN, the present device achieves this performance with a much lower operating voltage and a substantially higher current density, which is advantageous for direct-current output and practical energy delivery.

**Table 3:** Comparison of electrical output performance of representative PVDF-based TENGs

Tribo-layer material	Voltage (V)	Current density (µA cm^−2^)	Power density (µW cm^−2^)	Ref
PVDF/Graphene	1511	18,9	13.02	48
P(VDF-*co*-HFP)/MXene	225	11	168	49
P(VDF-TrFE)/SiO_2_	20	3.2	6.2	50
PVDF/PAN	160	60	199	51
PVDF/PP	29.7	2.4	9.58	52
PVDF/Cellulose Filter Paper	334	4,36	5.27	53
PVDF/CFO	17.2	2.27	9.03	54
P–Ti80	0.7	230	40.8	This work
P–Si–Ti120	0.72	341	82.8	This work

The long-term cycling data further confirms the operational durability of the optimized device. As shown in Fig. 8b, the P–Si–Ti120 TENG maintains stable performance over more than 4000 working cycles, with the output decreasing by only about 3%. This result indicates that the membrane can withstand repeated mechanical deformation without severe degradation of the internal transport pathways or interfacial charge-extraction behavior. Such stability is particularly important for porous and humidity-activated polymer membranes, which are often susceptible to structural relaxation and performance loss during prolonged operation. The excellent cycling durability therefore supports the beneficial role of VPI in reinforcing the membrane framework and preserving stable electrical output under repeated use.^38^Fig. 8c shows the direct charging behavior of a single TENG cell toward capacitors with different capacitances. The charging time depends strongly on the capacitance value: to reach a voltage of approximately 0.6 V, the device requires about 2 s, 15 s, and 50 s for the three capacitors, respectively. This trend is expected because a larger capacitor requires more charge to reach the same voltage. Fig. 8d further demonstrates the scalability of the device through simple series integration. When four TENG cells are connected in series, the output voltage increases markedly and can reach as high as 2.38 V. The practical significance of this voltage enhancement is further illustrated in Fig. 8e, where the four-cell configuration directly charges a 470 µF capacitor to about 1.8 V within 150 s. The inset photographs further confirm the practical utility of the stored energy, showing that the charged capacitor can successfully power a digital thermo-hygrometer (HTC-1). These results highlight the potential of the P–Si–Ti120 TENG system for high-current energy harvesting and self-powered humidity-responsive electronics.

## 4 Conclusions

In summary, we developed a DC-TENG based on electrospun porous PVDF fibers membrane through the combined use of SiO_2_-VPI and TiO_2_-ALD. The results show that VPI does not significantly alter the fibers morphology or crystalline phase structure of PVDF, but it improves membrane wettability, introduces internal inorganic functionality, and creates a more hydration-active transport environment. This internal activation also promotes more uniform subsequent TiO_2_ growth, allowing the VPI treatment series to outperform the directly coated controls at matched numbers of deposition cycles. As a result, the optimized P–Si–Ti120 TENG achieves a current density of 341 µA cm^−2^, an output voltage of 0.72 V, and a maximum power density of 82.8 µW cm^−2^, clearly exceeding the performance of the P–Ti80 TENG. Mechanistic analysis indicates that the enhanced output originates from the cooperative action of humidity-assisted proton/ion transport within the membrane and Schottky-regulated carrier extraction at the TiO_2_/metal interface. In addition to high electrical performance, the device shows good cycling stability over more than 4000 cycles, direct capacitor charging without an external rectifier, and practical capability to power a small digital thermo-hygrometer after energy storage. These findings establish the combination of SiO_2-_VPI and TiO_2_-SALD as an effective dual-scale engineering strategy for converting porous PVDF fibers membrane into robust and efficient DC-TENG platforms for self-powered electronics and humidity-responsive energy harvesting.

## Author contributions

Duy Linh Vu: writing – original draft, writing – review and editing, supervision, project administration, conceptualization, methodology, investigation. Thi Thuong Nguyen: writing – original draft, methodology, investigation, formal analysis, data curation. Dinh Nam Nguyen: validation, investigation, formal analysis, data curation. Hung-Anh Tran Vu: investigation, data curation. Ha Thi Vu Nguyen: investigation, formal analysis, formal analysis. Quang Tan Nguyen: writing – review and editing, methodology, formal analysis, conceptualization. Nguyen Xuan Duong: writing – review and editing, visualization, validation. Ngoc Thanh Duong: writing – review and editing, methodology, visualization. Minh Hieu Nguyen: writing – review and editing, conceptualization, data curation. Viet Huong Nguyen: writing – review and editing, supervision, visualization.

## Conflicts of interest

The authors declare that they have no conflict of interest.

## Supplementary Material

RA-016-D6RA03149H-s001

## Data Availability

The data presented in this study are available upon reasonable request from the corresponding author. Supplementary information (SI) is available. See DOI: https://doi.org/10.1039/d6ra03149h.
