# Supplementary material for: Enhanced performance of direct-current triboelectric nanogenerators based on SiO2-PVDF composite fibers coated with TiO2via spatial atomic layer deposition
Source: RSC Adv. 2026 Jul 3;16(35):35601–12. doi: 10.1039/d6ra03149h (PMC13329820; doi:10.1039/d6ra03149h)
Supplement: RA-016-D6RA03149H-s001 [file RA-016-D6RA03149H-s001.pdf]

Supplementary Information (SI) for RSC Advances.
This journal is © The Royal Society of Chemistry 2026

Supplementary Information

Enhanced Performance of Direct-Current Triboelectric Nanogenerators based on SiO_2-PVDF Composite Fibers Coated with TiO_2 via Spatial Atomic Layer Deposition

Duy Linh Vu,[*a] Thi Thuong Nguyen,[a] Dinh Nam Nguyen,[a] Hung-Anh Tran Vu,[a] Ha Thi Vu Nguyen,[a] Quang Tan Nguyen,[b] Nguyen Xuan Duong,[c] Ngoc Thanh Duong,[a] Hieu Minh Nguyen,[a] Viet Huong Nguyen,[*a]

[a] *Faculty of Materials Science and Engineering, Phenikaa School of Engineering, Phenikaa University, Hanoi 12116, Vietnam*

[b] *Center for Environmental Intelligence, VinUniversity, Hanoi 100000, Vietnam*

[c] *Faculty of Electronics and Telecommunications, VNU University of Engineering and Technology, Vietnam National University, Hanoi 12116, Vietnam*

∗ Corresponding author.

E-mail address:
linh.vuduy@phenikaa-uni.edu.vn, Duy Linh Vu
huong.nguyenviet@phenikaa-uni.edu.vn, Viet Huong Nguyen

I–V characteristic curves: The I–V characteristics of the membrane with the electrode pair were characterized using a potentiostat (CS350M EIS Potentiostat). Prior to measurement, the membrane was conditioned at 100% RH for 6 h to ensure complete water absorption. During measurement, two electrodes (1 × 1 cm) were placed on opposite sides of the membrane, and a voltage sweep from –1 V to 1 V was applied while recording the corresponding current response.

The rectification ratio calculated with the range of -1 V to 1 V is experessed by:

$$R_f = \frac{|I(1V)|}{|I(-1V)|} \#(S1)$$

Dry state:

$$R_f = \frac{|0.00639|}{|0.00281|} = 2.27$$

Wet state:

$$R_f = \frac{|0.4027|}{|0.00345|} = 116.72$$

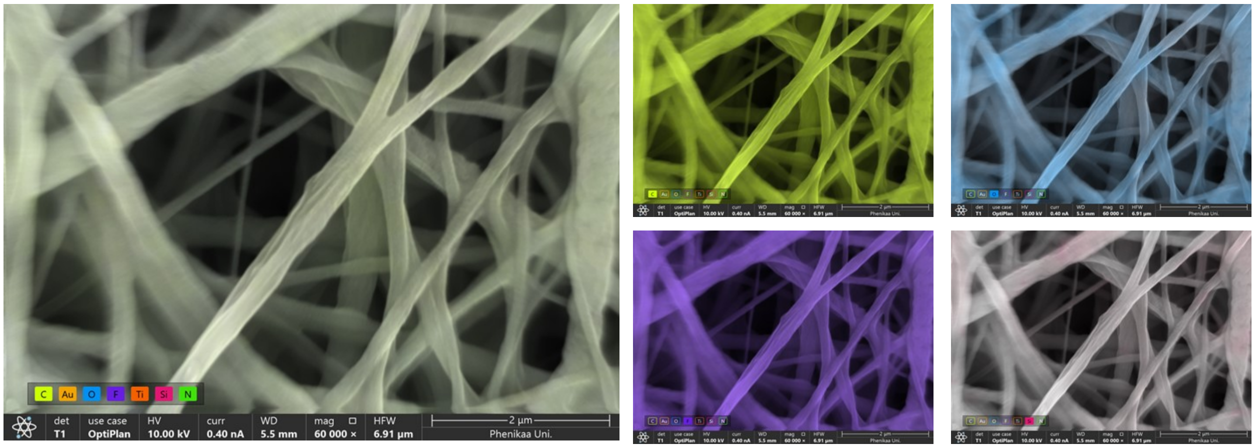

Figure S1. EDS QuantMap analysis of the SiO_2-PVDF composite fibers membrane

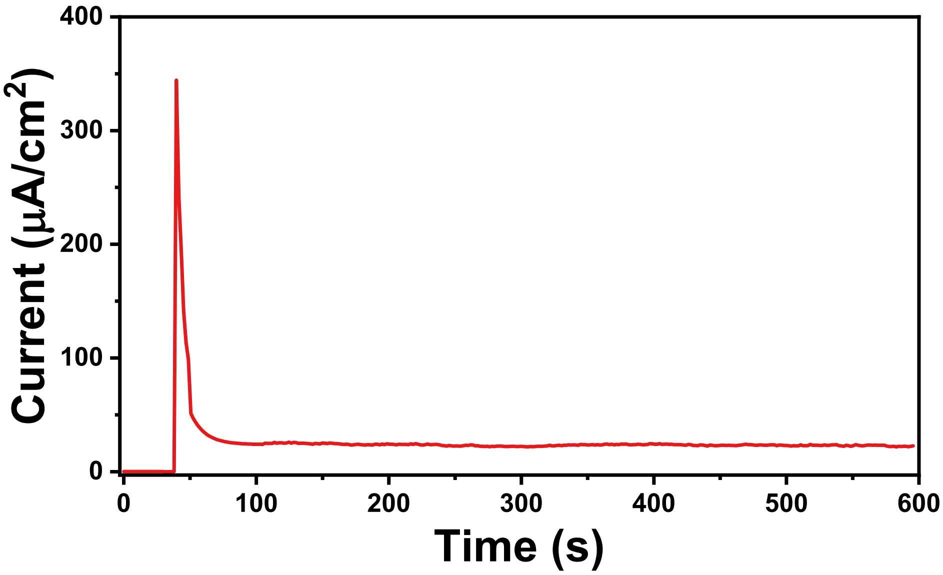

Figure S2. Static-current control measurement of the humidified device after electrode contact without further mechanical motion.
